# Combinatory effects of siRNA‐induced myostatin inhibition and exercise on skeletal muscle homeostasis and body composition

**DOI:** 10.1002/phy2.262

**Published:** 2014-03-20

**Authors:** Stephanie Mosler, Karima Relizani, Etienne Mouisel, Helge Amthor, Patrick Diel

**Affiliations:** ^1^ Department of Molecular and Cellular Sports Medicine German Sport University Cologne Cologne Germany; ^2^ Université Pierre et Marie Curie Institut de Myologie Unité mixte de recherche UPMC‐AIM UM 76 INSERM U 974 CNRS UMR 7215 75013 Paris France; ^3^ Department of Neuropediatrics and NeuroCure Clinical Research Center Charité Universitätsmedizin Berlin 13353 Berlin Germany; ^4^ Present address: Division of Sports‐ and Rehabilitation Medicine Ulm University Hospital Ulm Germany; ^5^ Present address: Obesity Research Laboratory Institute of Metabolic and Cardiovascular Diseases (I2MC) University Paul Sabatier – Inserm UMR 1048 Toulouse France; ^6^ Present address: Laboratoire Biothérapies des Maladies Neuromusculaires UFR des Sciences de la Santé Simone Veil Université de Versailles St‐Quentin‐en‐Yvelines 78180 Montigny‐le‐Bretonneux France

**Keywords:** Exercise, follistatin, myostatin, RNA interference

## Abstract

Inhibition of *myostatin* (*Mstn*) stimulates skeletal muscle growth, reduces body fat, and induces a number of metabolic changes. However, it remains unexplored how exercise training modulates the response to *Mstn* inhibition. The aim of this study was to investigate how siRNA‐mediated *Mstn* inhibition alone but also in combination with physical activity affects body composition and skeletal muscle homeostasis. Adult mice were treated with *Mstn*‐targeting siRNA and subjected to a treadmill‐based exercise protocol for 4 weeks. Effects on skeletal muscle and fat tissue, expression of genes, and serum concentration of proteins involved in myostatin signaling, skeletal muscle homeostasis, and lipid metabolism were investigated and compared with *Mstn* ^*−/−*^ mice. The combination of siRNA‐mediated *Mstn* knockdown and exercise induced skeletal muscle hypertrophy, which was associated with an upregulation of markers for satellite cell activity. SiRNA‐mediated *Mstn* knockdown decreased visceral fat and modulated lipid metabolism similar to effects observed in *Mstn* ^*−/−*^ mice. Myostatin did not regulate its own expression via an autoregulatory loop, however, *Mstn* knockdown resulted in a decrease in the serum concentrations of myostatin propeptide, leptin, and follistatin. The ratio of these three parameters was distinct between *Mstn* knockdown, exercise, and their combination. Taken together, siRNA‐mediated *Mstn* knockdown in combination with exercise stimulated skeletal muscle hypertrophy. Each intervention or their combination induced a specific set of adaptive responses in the skeletal muscle and fat metabolism which could be identified by marker proteins in serum.

## Introduction

During the last years, increasing interest focused on inhibiting the signal transduction of the muscle growth factor myostatin (*Mstn*) with the aim to develop strategies for the treatment of muscle disorders (Bradley et al. 2008; Tsuchida 2008), or metabolic diseases such as type II diabetes or adiposity (McPherron 2010).

Myostatin is a member of the transforming growth factor *β* (TGF‐*β*) family of signaling molecules, which negatively regulates muscle growth and differentiation (McPherron et al. 1997). As described for *Mstn* knockout mouse (*Mstn* ^*−/−*^), the absence of myostatin results in increased skeletal muscle mass, reduced fat tissue, and increased insulin sensitivity (McPherron and Lee 2002; Guo et al. 2009). Subsequently, a number of strategies were developed to block the effect of myostatin and tested on various models for neuromuscular disorders, muscle wasting conditions, or metabolic disturbances (Amthor and Hoogaars 2012). RNA interference (RNAi) has also been used to inhibit myostatin signaling (Kinouchi et al. 2008; Liu et al. 2008). This strategy is based on small interfering RNAs (siRNAs) which bind to their specific target mRNA sequence and induce cleavage of the mRNA with the consequence of “silencing” the target gene. Several research groups showed that gene knockdown of *Mstn* by RNAi is a promising therapeutic strategy for muscle wasting disorders (Acosta et al. 2005; Magee et al. 2006). Kinouchi et al. (2008), Liu et al. (2008) and have shown that efficient knockdown of *Mstn* resulted in increased skeletal muscle mass in mice following intravenous as well as oral application of *Mstn*‐specific siRNAs. So far, the role of exercise training in the siRNA‐induced MSTN inhibition is not described; a situation which might be relevant for the improvement of therapeutic options, but also in athletes as potential doping strategy. It is likely that the combination of myostatin blockade and exercise may induce synergistic effects. We hypothesize that synergistic effects from myostatin blockade and exercise may improve the therapeutic benefit of myostatin blockade in muscle disorders and metabolic diseases. However, such synergistic effects may also be abused by athletes as a potential doping strategy. Scientific information regarding such combinatory effects is limited. Therefore, the major aim of this study was to investigate in an animal model in mice how myostatin inhibition using *Mstn*‐targeting siRNA in combination with physical activity affects muscle growth, body composition, and metabolism.

## Materials and Methods

### Animals, training, and experimental treatments

8‐week‐old female Balb/c mice were purchased (Janvier, Le‐Genest St‐Isle, France) and acclimatized for 1 week before starting experiments. The mice were kept under controlled conditions (temperature 20 ± 1°C, humidity 50–80%, illumination 12L/12D) and had free access to water and a diet low in phytoestrogen content (R/M‐H, Ssniff GmbH, Soest, Germany). Mice were maintained according to the European Union guidelines for the care and use of laboratory animals. The study was undertaken with the approval of the regional administration of the governmental body. *Mstn* ^*−/−*^ founder breeding pairs on a C57BL/6 background were a kind gift from Se‐Jin Lee (McPherron et al. 1997). Muscles and serum from 4–5‐month‐old female *Mstn* ^*−/−*^ mice and *Mstn* ^*+/+*^ were obtained following sacrifice.

### siRNA treatment

siRNA‐targeting *Mstn* was custom‐made (Qiagen, Hilden, Germany). siRNA sequences were used as previously published (GDF8 siRNA26, 5′‐AAGATGACGATTATCACGCTA‐3′, position 426–446) (Magee et al. 2006; Kinouchi et al. 2008). The lyophilized siRNA was resuspended in sterile phosphate‐buffered saline (Dulbecco's Phosphate Buffered Saline (D‐PBS), Invitrogen, Karlsruhe, Germany) and injected using osmotic mini pumps (pump model 2006, Cat.‐No. 0007223, ALZET^®^ Osmotic Pumps, Cupertino, Canada). Using a flow rate of 0.15 *μ*L/h, 100 nmol/L/kg/day (equivalent of 1.5 mg/kg/day) siRNA was applied during a period of 28 days.

**Figure 1:**
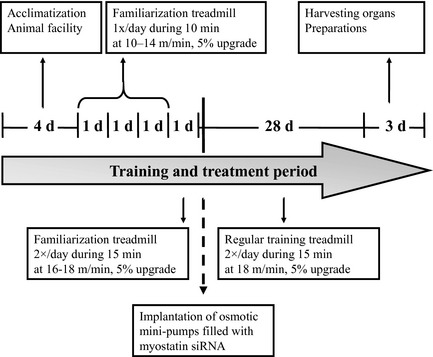
Schematic overview of study design.

The mice were randomly allocated to treatment or training groups (*n* = 7 animals per group). Mice were exercised on a motor‐driven rodent treadmill (Columbus Instruments, Columbus, OH) for 5 days/week over 4 weeks at 5% upgrade declination. The exercise intensity was progressively increased from 10 min once a day at 10 m/min to 15 min twice a day at 18 m/min during the first week. The study design is illustrated in Fig. 1.

### Production and injection of AAV‐propeptide for myostatin blockade

The myostatin propeptide construct was prepared by PCR amplification of C57BL/6 cDNA, using the oligonucleotide primers 5′‐CCG CTC GAG ATG ATG CAA AAA CTG CAA ATG‐3′ and 5′‐CCG GGA TCC CTA TTA GTC TCT CCG GGA CCT CTT‐3′ and was introduced into an AAV2‐based vector between the two inverted terminal repeats and under the control of the cytomegalovirus promoter using the XhoI and BamHI restriction enzyme sites. The AAV myostatin propeptide was produced in human embryonic kidney (HEK) 293 cells by the triple‐transfection method using the calcium phosphate precipitation technique with both the pAAV2 propeptide plasmid, the pXX6 plasmid coding for the adenoviral sequences essential for AAV production, and the pRepCAp plasmid coding for the AAV1 capsid. The virus was then purified by two cycles of cesium chloride gradient centrifugation and concentrated by dialysis. The final viral preparations were kept in PBS solution at −80°C. The particle titer (number of viral genomes) was determined by a quantitative PCR. A volume of 50 *μ*L of AAV2/1‐myostatin propeptide (5 × 10^11^ vg) or control AAV (5 × 10^11^ vg of AAV2/1‐U7‐scramble) were injected into the *tibialis anterior* (*TA*) muscles of 2‐month‐old C57Bl/6 mice. *TA* muscles were dissected following cervical dislocation of mice 1 month after intramuscular injection of AAV2/1‐propeptide.

### Tissue collection and preparation

At the end of the exercise protocol, body weights of the mice were determined and animals sacrificed. Blood samples were collected and centrifuged, and serum cryoconserved. Following dissection, wet weights of liver, visceral fat, and *gastrocnemius* muscles were determined. Muscles were snap‐frozen in liquid nitrogen or mounted for histological analysis.

### RNA isolation and real‐time RT‐PCR

Total RNA was isolated from pooled frozen tissues by the method of Chomczynski and Sacchi (Chomczynski and Sacchi 1978) using Trizol^®^ (Invitrogen) followed by first‐strand cDNA synthesis (QuantiTect Rev. Transcription Kit, Qiagen, Hilden, Germany). Real‐time q‐PCR was performed in a MX3005P thermal cycler (Stratagene, Agilent Technologies, Santa Clara, CA). The protocol comprised 4 min at 95°C followed by 45 cycles of 95, 58, and 72°C for 30 sec each. Based on the cDNA sequences available at the EMBL database, the specific primer pairs for *Cyclophilin*,* Mstn*,* Fst*,* MyoD*,* Pax‐7* were designed by the software primer3 (Whitehead Institute for Biomedical Research, Cambridge, MA; http://www-genome.wi.mit.edu/cgi-bin/primer/primer3_www.cgi/) and confirmed by the sequences in the NCBI database (http://www.ncbi.nlm.nih.gov/). All primers were synthesized by Invitrogen. The primer pairs are listed in Table 1. The data were normalized to the *Cyclophilin* expression as a reference gene using the ΔΔCt method and the relative expression levels of the genes are reported as the fold induction (Livak and Schmittgen 2001; Pfaffl 2001; Velders et al. 2012).

**Table 1:** Primer sequences

Primer	Direction	Sequences
*Cyclophilin*	Fwd	5′‐ GGATTCATGTGCCAGGGTGG‐3′
	Rev	5′‐ CACATGCTTGCCATCCAGCC ‐3′
*Fst*	Fwd	5΄‐ CCGCCACACTGGATATCTTC ‐3΄
	Rev	5΄‐ CCGCCACACTGGATATCTTC ‐3΄
*Mstn*	Fwd	5′‐TAACCTTCCCAGGACCAGGA‐3′
	Rev	5′‐CACTCTCCAGAGCAGTAATT‐3′
*MyoD*	Fwd	5′‐CGGCTACCCAAGGTGGAGAT‐3′
	Rev	5′‐GAGCACTCGGCTAATCGAAC‐3′
*Pax‐7*	Fwd	5′‐CCGTGTTTCTCATGGTTGTG‐3′
	Rev	5′‐GAGCACTCGGCTAATCGAAC‐3′
*Mstn* (Fig. 5A and C)	Fwd	5′‐TAACCTTCCCAGGACCAGGAG‐3′
	Rev	5′‐GCAGTCAAGCCCAAAGTCTC‐3′
*Mstn‐propeptide*	Fwd	5′‐TGACAGCAGTGATGGCTCTT‐3′
	Rev	5′‐CCGTCTTTCATGGGTTTGAT‐3′

Real‐Time qPCR for Fig. 5A–D was performed according to the SYBR Green^®^ protocol (Applied Biosystems). Total RNA was isolated from frozen muscles after pulverization in liquid nitrogen with the Trizol^®^ (Invitrogen) extraction protocol. Isolated RNA was quantified using the NanoDrop^®^ ND‐1000 spectrophotometer (Thermo scientific, Waltham, MA) and cDNA was synthesized using the Thermoscript^®^ RT PCR System (Invitrogen). After cDNA synthesis, Real‐Time PCR was performed by using the SYBR Green^®^ PCR Master Mix Protocol (Applied Biosystems, Madrid, Spain) in triplicate on The ECO Real‐Time PCR System (Illumina, Little Chesterford, Essex, U.K.) with a hotstart Taq polymerase. A 10‐min denaturation step at 94°C was followed by 40 cycles of denaturation at 94°C for 10 sec and annealing/extension at 60°C for 30 sec. Before sample analysis, we had determined for each gene the PCR efficiencies with a standard dilution series (10^0^–10^7^ copies/*μ*L), which subsequently enabled us to calculate the copy numbers from the *C*_t_ values (Pfaffl 2001). mRNA levels were normalized to 18S rRNA.

### Quantification of serum follistatin and myostatin propeptide

Serum levels of follistatin and myostatin propeptide were determined by Immuno‐PCR‐based assay using (Chimera Imperacer^®^ kit [Chimera Biotec GmbH, Dortmund, Germany], 11‐000 kit‐R and 11‐039 kit‐R). The following capture antibodies and recombinant proteins were used: goat polyclonal antihuman follistatin antibody (AF669, R&D Systems GmbH, Wiesbaden‐Nordenstadt, Germany), chicken polyclonal antihuman myostatin propeptide antibody (RD183057050, BioVendor), recombinant human follistatin (669FO/CF, R&D Systems), recombinant human myostatin propeptide (RD172058100, BioVendor GmbH, Heidelberg, Germany). For spiking, standardized serum was used (BISEKO, Biotest AG, Dreieich, Germany) for follistatin and sample dilution buffer (SDB2000) for myostatin propeptide. Immuno‐PCR was performed as described in Diel et al. (2010).

### Quantification of serum leptin

Leptin concentrations were determined using the ELISA method (mouse‐/rat‐leptin ELISA E06 kit, Mediagnost GmbH, Reutlingen, Germany). The analytical sensitivity of the assay was 0.01 ng/mL and the intra‐ and interassay variance was ≤5%. Serum samples were diluted 1:5 in the provided dilution buffer (VP) and the assay was conducted according to the manufacturer's protocol.

### Quantification of serum lipids and liver triglycerides

Serum levels of cholesterol and high‐density lipoprotein cholesterol (HDL) were determined using photometry (DIALAB, Wiener Neudorf, Austria). Serum and liver triglycerides were analyzed using colorimetry (ABX Pentra; ABX Diagnostics, Montpellier, France). For determination of liver triglyceride content, 100 mg of liver tissue was powdered in liquid nitrogen, then incubated for 1 h at 4°C in lysis buffer (50 mmol/L Tris, pH 8.0, 2 mmol/L CaCl_2,_ 80 mmol/L NaCL and 1% Triton x‐100) in presence of enzyme inhibitor PMSF (phenylmethanesulfonyl fluoride, dissolved in isopropanol) at a final concentration of 10 mmol/L. The solution was then centrifuged at 8000 rpm for 20 min at 4°C and protein concentration determined (DC Protein Assay; Bio‐Rad, München, Germany). The triglyceride content was determined as described above and referred to the protein content (mmol triglycerides/g protein).

### Histological analysis

Transverse sections (7 *μ*m) were cut from the mid belly region of *gastrocnemius* muscle using a cryostat (Leica, Wetzlar, Germany, CM 1510S) and were then mounted on slides coated with polylysine (Menzel Gläser, Hilden, Germany). Cryo‐sections were stained with hematoxylin and eosin and images acquired with a light microscope (Axiophot, Zeiss, Jena, Germany). Myofiber cross‐sectional area was determined using the ImageJ 1.33 program software (National Institute of Health, http://rsb.info.nih.gov/ij/); 80–120 myofibers per muscle were analyzed (*n* = 7 animals each group).

### Statistical analysis

All data are presented as means ± standard deviation (SD). A two‐way Mann–Whitney *U*‐Test was performed for comparison between two groups. For statistical analysis of more than two groups, data were calculated using a Kruskal–Wallis *H*‐test followed by Mann–Whitney *U*‐test. Significance levels were set at *P* < 0.05.

## Results

### siRNA targeted to *Mstn* increased muscles mass

**Figure 2:**
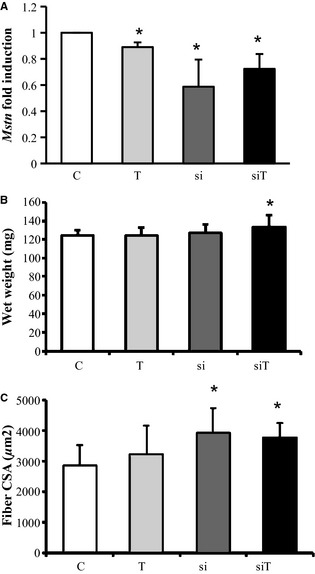
Effect of *Mstn* knockdown and exercise on muscle morphometry and *Mstn* mRNA expression of *gastrocnemius* muscle. (A) Quantitative RT‐PCR analysis of *Mstn* mRNA levels following siRNA‐mediated *Mstn* knockdown ± exercise. (B) Muscle wet weight following siRNA‐mediated *Mstn* knockdown ± exercise. (C) Fiber cross‐sectional area (CSA) following siRNA‐mediated *Mstn* knockdown ± exercise. C = control group, T = training group, si = treatment with siRNA, siT = training + siRNA. KO = *Mstn* ^*−/−*^ mice, WT = wild‐type mice. Values are presented as means ± SD. *n* = 7 per group. *P < 0.05 significantly different from the control group.

We systemically treated adult wild‐type mice with continuous *Mstn* siRNA or PBS perfusions for 28 days by osmotic minipumps. Both treatment groups (±*Mstn* siRNA) were further subdivided and either subjected to a treadmill training program or to sedentary condition, resulting in four different experimental conditions: (1) non‐siRNA‐treated/nontrained control mice (named C), (2) non‐siRNA‐treated/trained mice (named T), (3) siRNA‐treated/nontrained mice (named si), and (4) siRNA‐treated/trained mice (named siT). We used a *Mstn* siRNA sequence that previously proved very efficient to block the effect of *Mstn* (Magee et al. 2006; Kinouchi et al. 2008). In agreement, we here confirm efficient gene knockdown of *Mstn* in *gastrocnemius* muscle in both exercised and nonexercised animals (Fig. 2A). Interestingly, *Mstn* was also downregulated in non‐siRNA‐treated/exercised muscle, showing an effect of exercise on *Mstn* regulation. However, exercise together with siRNA treatment had no synergistic effect on gene knockdown. Combination of exercise and systemic treatment with *Mstn* siRNA for 28 days significantly stimulated muscle growth as shown for the *gastrocnemius* muscle, whereas treatment with *Mstn* siRNA on its own did not result in remarkably changes in the wet weight of this muscle. Nevertheless, gastrocnemius muscle weight was higher in the siRNA group compared to the control and training group (mean weight: 127 mg in si compared to 124 mg in *C* and *T*). Furthermore, exercise on its own had no effect on muscle mass (Fig. 2B). In order to determine the effects of *Mstn*‐targeting siRNA and exercise on *gastrocnemius* muscle fibers, we measured the fiber cross‐sectional area (CSA) and found a significant shift toward larger fibers in both siRNA‐treated animal groups compared to the control animals, proving a hypertrophic growth response at individual myofiber level (Fig. 2C).

### Effects of *Mstn* siRNA treatment on target genes involved in skeletal muscle adaptation

**Figure 3:**
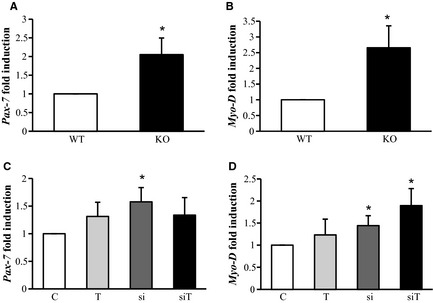
Effect of Mstn knockdown and training on relative mRNA expression of *Pax7* and *MyoD* in gastrocnemius muscle. (A) Quantitative RT‐PCR analysis of *Pax7* mRNA levels in *Mstn^−/−^* muscle. (B) Quantitative RT‐PCR analysis of MyoD mRNA levels in *Mstn^−/−^* muscle. (C) Quantitative RT‐PCR analysis of Pax7 mRNA levels following siRNA‐mediated Mstn knockdown ± exercise. (D) Quantitative RT‐PCR analysis of *MyoD* mRNA levels following siRNA mediated *Mstn* knockdown ± exercise. C = control group, T = training group, si = treatment with siRNA, siT = training + siRNA. KO = *Mstn^−/−^* mice, WT = wild‐type mice. Values are presented as means ± SD. *n* = 7 per group. *P < 0.05 significantly different from control group.

Previous works on the mechanism of muscle growth in lack of *Mstn* evidenced an activation of muscle satellite cells (McCroskery et al. 2003; Wang and McPherron 2012). In agreement, we here show elevated transcript levels for *Pax‐7* and *MyoD* in skeletal muscle from *Mstn* ^*−/−*^ mice (Fig. 3A and B), allowing for a molecular read‐out of the effect of siRNA‐mediated *Mstn* knockdown on satellite cells. Similar as for *Mstn* ^*−/−*^ mice, siRNA‐mediated *Mstn* knockdown resulted in an upregulation of *Pax‐7* expression (Fig. 3C). MyoD was significantly increased after siRNA treatment. Also, training resulted in a slight increase in MyoD expression. Interestingly, the combination of both further increased MyoD expression in an adaptive (Fig. 3D).

### Mstn siRNA reduces visceral body fat content and improves serum lipid levels

**Figure 4:**
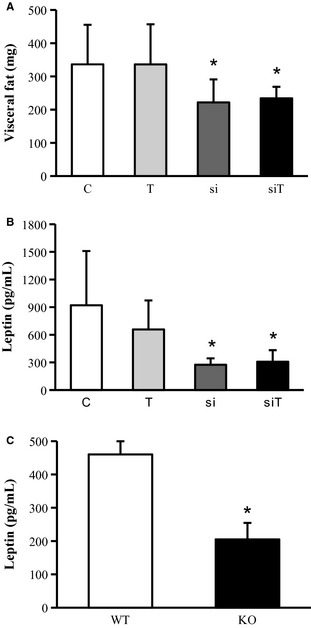
Effects of *Mstn* knockdown and exercise on visceral body fat content and serum levels of leptin. (A) Visceral fat mass following siRNA‐mediated *Mstn* knockdown. (B) Serum levels of leptin following siRNA‐mediated *Mstn* knockdown. (C) Serum levels of leptin in *Mstn* ^*−/−*^ mice. C = control group, T = training group, si = treatment with siRNA, siT = training + siRNA. KO = *Mstn* ^*−/−*^ mice, WT = wild‐type mice. Values are presented as means ± SD. *n* = 7 per group *P < 0.05 significantly different from control group.

Deletion or blockade of myostatin results in decreased body fat (Guo et al. 2009). Accordingly, we here show that visceral fat was significantly reduced in both *Mstn* siRNA‐treated groups (Fig. 4A). The decreased fat tissue entailed reduced serum leptin levels (Fig. 4B; Table 3). Similar reduced serum leptin was also found in *Mstn* ^*−/−*^ mice and confirms previously published data (Fig. 4C) (Guo et al. 2009).

Associated with loss in fat tissue, HDL cholesterol was significantly increased in response to training, siRNA treatment, and by the combination of both, whereas serum triglycerides were significantly decreased (Table 2). Interestingly, total cholesterol was elevated following combination of siRNA treatment and exercise, which was associated with a strongly increased HDL value and little effect on serum triglycerides. Furthermore, in both siRNA‐treated groups (si and siT), we observed a tendency for decreased liver triglyceride levels, which statistically remained insignificant in comparison with the control group. Interestingly, exercise alone strongly increased liver triglycerides (Table 2).

**Table 2:** Serum lipids and liver triglycerides

	Control (*C*)	Training (*T*)	siRNA (si)	siRNA + Training (siT)
Cholesterol (mg/dL)	82.83 ± 5.42	86.85 ± 6.84	83 ± 11.32	100.67 ± 7.00^a^
HDL (mg/dL)	42.5 ± 30.95	75 ± 8.16^a^	69.42 ± 11.47^a^	84 ± 5.83^a^
Serum triglycerides (mg/dL)	163.7 ± 27.8	119 ± 14.4^a^	138.3 ± 35.5^a^	161.3 ± 32.5
Liver triglycerides (mmol/g)	0.021 ± 0.003	0.032 ± 0.011^a^	0.017 ± 0.04	0.017 ± 0.05

Serum lipid and liver triglyceride levels after the 4‐weeks treatment (s. c. application of 100 nmol/L Myostatin siRNA/kg/bw/day via osmotic minipumps) and training period. *n* = 7. Data shown are means ± SD.

a *P* ≤ 0.05, significant different from control group.

### The effect of myostatin blockade on the expression of myostatin and myostatin propeptide

Having established that myostatin blockade in combination with exercise resulted in profound changes in skeletal muscle homeostasis and body metabolism, we now questioned whether knockdown of myostatin results in feedback loops and changes in expression of myostatin and myostatin‐binding proteins.

We first investigated whether interference with myostatin signaling impacts its own expression.

**Figure 5:**
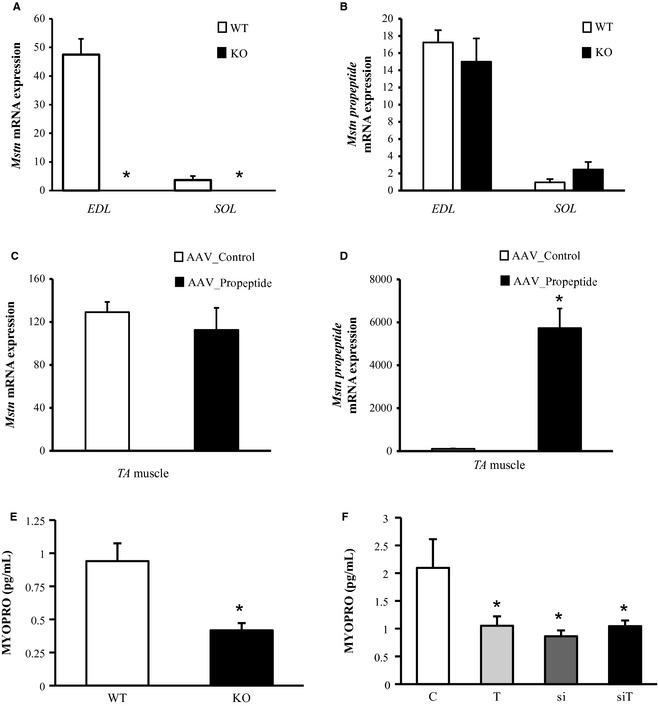
Effect of myostatin inhibition (after myostatin knockout [*Mstn* ^*−/−*^], AAV‐mediated overexpression of myostatin propeptide [AAV Prop] and siRNA‐mediated *Mstn* knockdown) on myostatin propeptide mRNA levels and serum protein levels. (A) Quantitative RT‐PCR analysis of *Mstn* mRNA levels in *Mstn* ^*−/−*^ *extensor digitorum longus* (*EDL*) and *soleus* muscles (primers targeting exons 2/3). *n* = 5 per group, *P < 0.05 (B) Quantitative RT‐PCR analysis of *myostatin propeptide* mRNA levels in *Mstn* ^*−/−*^ muscle (primers targeting exons 1/2). *n* = 5 per group (C) Quantitative RT‐PCR analysis of *Mstn* mRNA levels in AAV Propeptide treated *tibialis anterior* (*TA*) muscle (primers targeting exons 2/3). *n* = 6 per group *P < 0.05 (D) Quantitative RT‐PCR analysis of *myostatin propeptide* RNA levels in AAV Propeptide‐treated *TA* muscle (primers targeting exon 1/2). *n* = 6 per group, *P < 0.05 (E) Immuno‐PCR analysis to determine serum concentration of myostatin propeptide (MYOPRO) from *Mstn* ^*−/−*^ mice. *n* = 5 per group, *P < 0.05. (F) Immuno‐PCR analysis to determine serum concentration of MYOPRO following siRNA‐mediated *Mstn* knockdown ± exercise. *n* = 7 per group, *P < 0.05 significantly different from control group. C = control group, T = training group, si = treatment with siRNA, siT = training + siRNA. KO = *Mstn* ^*−/−*^ mice, WT = wild‐type mice. Values are presented as means ± SD.

*Mstn* ^*−/−*^ mice consists of a deletion of *Mstn* exon 3, which is the c‐terminal fragment of the gene encoding the mature part of the myostatin protein, leaving intact the promoter and the myostatin propeptide except for its last 12 amino acids (McPherron et al. 1997). It is unknown, however, whether this enables synthesis of a functional myostatin propeptide. We constructed primers to the *propeptide* region and the c‐terminal fragment in order to analyze the two parts of the *Mstn* gene. As expected, knockout of *Mstn* exon 3 completely abolished expression of the c‐terminal part encoding the mature myostatin (5A). Surprisingly, *propeptide* expression remained unchanged (Fig. 5B), suggesting no feedback loop of myostatin on its own expression. This finding was further corroborated when transfecting *tibialis anterior* muscle of wild‐type mice with AAV‐propeptide. As expected, AAV‐propeptide massively induced expression of the *propeptide* transgene (Fig. 5D). The expression of the c‐terminal part of *Mstn*, however, remained unchanged, further evidence that propeptide mediated myostatin blockade does not feedback on *Mstn* expression (Fig. 5C). Interestingly, despite normal *propeptide* RNA transcript levels, serum myostatin propeptide concentrations were reduced in *Mstn* ^*−/−*^ mice (Fig. 5E), likely reflecting reduced protein assembly, defective secretion or an unstable protein in lack of the mature myostatin region. Likewise, serum myostatin propeptide concentration was also reduced following siRNA mediated *Mstn* knockdown (Fig. 5F). However, exercise as well as siRNA‐mediated *Mstn* knockdown in combination with exercise decreased *Mstn* mRNA expression alongside with reduced serum myostatin propeptide levels (Figs. 2A and 5F). It should be noted that *propeptide* as well as the c‐terminal fragment of the *Mstn* gene are highly expressed in *extensor digitorum longus* (*EDL*) muscle from wild‐type mice but at extremely low levels in *soleus* muscle (Fig. 5A and B). This has been previously attributed to the different fiber‐type composition of the two muscles, the *EDL* being predominantly composed of fast fibers, whereas *soleus* muscle contains an important part of slow fibers (Agbulut et al. 2003). Against this hypothesis, we here show that propeptide expression did not change in *EDL* and *soleus* muscle following *Mstn* knockout, although both muscles completely change fiber‐type composition from oxidative toward fast glycolytic fibers (Girgenrath et al. 2005; Amthor et al. 2007). Thus, myostatin expression is not fiber‐type‐dependent, but is an intrinsic property of specific muscles.

### The effect of myostatin blockade on the expression of follistatin

**Figure 6:**
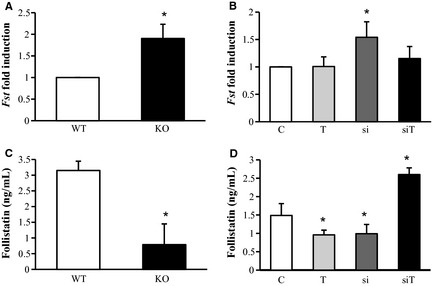
Effect of *Mstn* knockdown and training on *follistatin* mRNA expression of *gastrocnemius* muscle and serum protein level. (A) Quantitative RT‐PCR analysis of *follistatin* mRNA levels in *Mstn* ^*−/−*^ muscle. *n* = 5 per group, *P < 0.05 significantly different from WT. (B) Quantitative RT‐PCR analysis of *follistatin* mRNA levels following siRNA‐mediated *Mstn* knockdown ± exercise. *n* = 7 per group, *P < 0.05 significantly different from control group. (C) Immuno‐PCR analysis to determine serum concentration of follistatin from *Mstn* ^*−/−*^ mice. *n* = 5 per group, *P < 0.05 significantly different from WT. (D) Immuno‐PCR analysis to determine serum concentration of follistatin following siRNA‐mediated *Mstn* knockdown ± exercise. *n* = 7 per group, *P < 0.05 significantly different from control group. C = control group, T = training group, si = treatment with siRNA, siT = training + siRNA. KO = *Mstn* ^*−/−*^ mice, WT = wild‐type mice. Values are presented as means ± SD.

Follistatin is a strong modulator of myostatin activity as it physically interacts with myostatin thereby blocking its biological effect. Different follistatin isoforms result from alternative splicing (Inouye et al. 1991). The short isoform, FS288, binds heparan sulfate and locates to cellular surfaces, whereas the long isoform, FS315, is soluble and detected in serum (Inouye et al. 1992; Sugino et al. 1993; Schneyer et al. 2004). We next asked whether myostatin regulates the expression of its own antagonist follistatin. Indeed, *follistatin* mRNA expression was strongly upregulated in skeletal muscle from *Mstn* ^*−/−*^ mice (Fig. 6A) as well as following siRNA‐mediated *Mstn* knockdown (Fig. 6B). Importantly, exercise did not influence *follistatin* expression and when combined with *Mstn* siRNA, exercise completely prevented the stimulating effect on *follistatin* expression in skeletal muscle (Fig. 6B). To our surprise, the stimulating effect of *Mstn* knockdown on *follistatin* mRNA expression was not paralleled by increased serum follistatin protein levels. In fact, serum follistatin strongly decreased in *Mstn* ^*−/−*^ mice (Fig. 6C). Likewise, *Mstn* siRNA treatment caused decreased serum follistatin (Fig. 6D). However, exercise also decreased serum follistatin (Fig. 6D). Remarkably, combination of *Mstn* siRNA treatment and exercise strongly increased serum follistatin protein levels (Fig. 6D).

## Discussion

The purpose of this study was to characterize the combinatory effects of siRNA‐induced *Mstn* knockdown and physical training on molecular mechanisms involved in skeletal muscle adaptation, body composition, lipid metabolism, and myostatin‐interacting serum proteins.

We confirmed previous data showing that siRNA efficiently knocked down *Mstn* expression (Kinouchi et al. 2008) and in consequence induced a number of known effects of *Mstn* knockout, such as upregulation of the satellite cell markers, reduction in fat tissue, and decreased serum leptin (McPherron and Lee 2002; McCroskery et al. 2003). The induction of *Pax7* and *MyoD* expression strengthens previous findings on the use of *Mstn* siRNA (Liu et al. 2008), however, it is no proof for a recruitment of satellite cells during hypertrophic fiber growth. Recent data confirmed that satellite cells are recruited following myostatin blockade, however, this was rather a minor event and relatively late during the hypertrophic growth phase, therefore, only in part explaining the growth‐stimulating effect of myostatin blockade (Wang and McPherron 2012).

We hypothesized that the combination of myostatin blockade and exercise would result in synergistic effects. Those synergistic effects were observed for gastrocnemius muscle wet weight and MyoD mRNA expression. A likely explanation for the effect on muscle growth when the siRNA is used in the presence of exercise is the increase in serum follistatin which is a potent regulator of skeletal muscle hypertrophy. However, determination of CSA revealed that the additional training program in terms of strength training did not lead to further enhancement of the siRNA‐induced muscle hypertrophy. So far, some studies investigated the effects of endurance training in the absence of myostatin (Matsakas et al. 2010, 2012; Savage and McPherron 2010). Matsakas et al. (2010) identified that the muscle fiber hypertrophy, oxidative capacity, and glycolytic phenotype of myostatin‐deficient muscle can be altered with endurance exercise regimes. The authors observed that cross‐sectional area of hypertrophic myofibers from myostatin KO mice decreased toward wild‐type values in response to exercise. Anyway, the training regime in terms of swim training and wheel running increased muscle force in myostatin KO mice (Matsakas et al. 2012). In our study, a possible explanation for the missing additive effect of siRNA treatment on CSA when combined with training might be a self‐protection mechanism to protect the muscle against too strong hypertrophy. Such a self‐protection mechanism was already discussed in our observations with exercising rats treated with methandienone (Mosler et al. 2012). It is presumable that muscle hypertrophy can achieve only a distinct level when myostatin inhibition is combined with training to do not impair exercise performance. However, muscle performance such as grip strength or exercise performance was not examined in the context of this study. This issue might be worth to investigate in future.

Similar to *Mstn* siRNA, exercise also reduced *Mstn* expression, however, without entailing “typical” myostatin blockade effects, such as increased muscle mass, loss in fat, or changes in *Pax7*/*MyoD* transcription. In agreement, ample human and animal studies revealed that training downregulated *Mstn* mRNA contents in skeletal muscle (Roth et al. 2003; Matsakas et al. 2005; Mosler et al. 2012). Such reduced *Mstn* expression following exercise likely caused decreased serum myostatin propeptide levels. Reduced propeptide levels occurred also from *Mstn* knockdown in *Mstn* ^*−/−*^ mice as well as following treatment with *Mstn* siRNA. However, this must result from a different molecular mechanism, likely at posttranslational level, as transcription of the propeptide itself remained unchanged, an issue to be resolved in future work.

The effect of *Mstn* knockdown on follistatin expression was intriguing because *follistatin* transcription in skeletal muscle increased but serum follistatin protein decreased. As we cannot offer experimental insight into this apparent discrepancy, it is important to keep in mind, that different follistatin isoforms result from alternative splicing, giving rise to species that remain local or which are soluble (Inouye et al. 1992; Schneyer et al. 2004; Matsakas et al. 2005). The striking differences between muscle *follistatin* mRNA levels and serum follistatin protein levels following *Mstn* knockdown strongly suggests changes in alternative splicing of *follistatin* leading to higher local and lower soluble follistatin, a hypothesis that warrants further investigations. It remains to be determined whether myostatin blockade affects alternative splicing of *follistatin*. Such hypothesis offers an attractive explanation for the different effects of exercise, *Mstn* knockdown or the combination of both on serum follistatin. However, it is also possible that the differences between follistatin mRNA expression and protein expression in serum reflect any number of posttranslational differences in follistatin expression (including shifts in translational efficiency as well as follistatin degradation/stability).

In contrast to herein described results, previous studies did not reveal changes in serum myostatin propeptide and serum follistatin following physical training (Diel et al. 2010; Mosler et al. 2012). However, in these studies we analyzed the impact of endurance and strength training in male human subjects (Diel et al. 2010) and male rats after a 3‐week treadmill training (Mosler et al. 2012), but not the effects in females. As we here show that serum myostatin propeptide and serum follistatin concentrations were decreased after the 4‐week treadmill training in female mice (Figs. 5F and 6D; Table 3), a gender‐specific response to training in the analyzed serum markers seems to be possible. Indeed, in a previous study in humans, we detected gender differences in myostatin propeptide and follistatin concentrations (Mosler et al. 2013). Also McMahon et al. (2003) identified differences in myostatin serum concentration between males and females.

**Table 3:** Serum measurements of follistatin, myostatin propeptide, and leptin

	Follistatin	Myostatin propeptide	Leptin
Control	=	=	=
*Mstn* ^*−/−*^	↓	↓	↓
siRNA‐Mstn	↓	↓	↓
Physical training	↓	↓	=
siRNA‐Mstn + physical training	↑	↓	↓

Summary of serum measurements of follistatin, myostatin propeptide, and leptin in untreated sedentary mice (control), in *Mstn* ^*−/−*^ mice, following siRNA‐*Mstn* perfusion, following physical training and following combination of siRNA‐*Mstn* and physical training (values depicted in Figs. 4B and C, 5E and F, 6C and D. =: unchanged, ↑: up, ↓: down.

Synthetic antisense oligonucleotide chemistries are easy to synthesize and have already been tested in humans during clinical trials such as to induce exon skipping in Duchenne muscular dystrophy (Cirak et al. 2011; Goemans et al. 2011). We here show, supported by previous findings, that siRNA against myostatin can modulate skeletal muscle properties. This may, however, encourage misuse of *Mstn* knockdown strategies such as for doping purpose, and therefore requires development of novel detection methods. Importantly, such detection must be specific and independent of muscle activity and trained status. We here show that combination of different serum markers permit a specific signature of *Mstn* knockdown (decrease in serum follistatin, propeptide, and leptin). In Table 3, we summarize finding on serum measurements of follistatin, myostatin propeptide, and leptin (values are depicted in Figs. 4B and C, 5E and F, 6C and D). Importantly, we show that exercise mimicked *Mstn* knockdown in some respects (decrease in serum follistatin and propeptide), but not in others (unchanged serum leptin), whereas combination of exercise and *Mstn* knockdown was again different (increased serum follistatin, decreased propeptide and leptin). In conclusion, use of the three serum markers follistatin, propeptide and leptin possibly enables to differentiate four conditions: (1) sedentary condition, (2) sedentary condition in combination with *Mstn* knockdown, (3) exercise, and (4) exercise in combination with *Mstn* knockdown. However, the observed profile may be specific to the type and volume of exercise as well as the dose of MSTN inhibitor used in this study. Further, the profile also may be different in humans. At this time, such a serum marker profile might be a first approach for detection of indirect myostatin manipulations by siRNA.

It has previously been described that *Mstn* knockout improves serum lipid levels and liver triglycerides (McPherron and Lee 2002; Guo et al. 2009; McPherron 2010). Here, we support these findings and show that siRNA‐mediated *Mstn* knockdown also decreased serum and liver triglycerides (Table 2). Additionally, exercise training also resulted in decreased serum triglycerides, which is in line with results obtained in humans (Martin et al. 1997). However, when combined with siRNA treatment, the training effect was reversed. Interestingly, in the liver, exercise training remarkably increased triglyceride concentrations (Table 2), supporting recent results by Cambri et al. (2011). The authors presume that the release of free fatty acids by lipolysis exceeds the oxidation capacity of active muscles during prolonged physical exercise (Magkos et al. 2007); in consequence the remaining free fatty acids can be re‐esterified in the liver (Magkos et al. 2007; Cambri et al. 2011). The significant increase in serum HDL following siRNA‐*Mstn* treatment and exercise is a further beneficial effect of lipid metabolism and support previous findings on the effect of exercise training (Haskell 1984).

In summary, our data show that the siRNA‐mediated *Mstn* knockdown is an efficient myostatin blockade strategy. Especially in combination with physical training, myostatin blockade may offer new therapeutic options as well as has implications for antidoping research. *Mstn* knockdown also caused metabolic changes as indicated by the decreased visceral fat content, decreased serum leptin levels alongside with improved serum lipid status. This supports the use of myostatin blockade for the treatment of metabolic diseases such as type II diabetes or for obesity.

## Conflict of Interest

None declared.
